# Seroprevalence of severe acute respiratory syndrome coronavirus-2 (SARS-CoV-2) infection among Veterans Affairs healthcare system employees suggests higher risk of infection when exposed to SARS-CoV-2 outside the work environment

**DOI:** 10.1017/ice.2020.1220

**Published:** 2020-09-23

**Authors:** Derek E. Dimcheff, Richard J. Schildhouse, Mark S. Hausman, Brenda M. Vincent, Erica Markovitz, Stephen W. Chensue, Jane Deng, Melissa McLeod, Danielle Hagan, Jon Russell, Suzanne F. Bradley

**Affiliations:** 1 Hospital Medicine Section, Medicine Service, Veterans Affairs Ann Arbor Healthcare System, Ann Arbor, Michigan; 2 Division of Hospital Medicine, University of Michigan Medical School, Ann Arbor, Michigan; 3 Anesthesiology Service, Veterans Affairs Ann Arbor Healthcare System, Ann Arbor, Michigan; 4 Department of Anesthesia, University of Michigan Medical School, Ann Arbor, Michigan; 5 Center for Clinical Management Research, Veterans Affairs Ann Arbor Healthcare System, Ann Arbor, Michigan; 6 Primary Care Section, Ambulatory Care Service, Veterans Affairs Ann Arbor Healthcare System, Ann Arbor, Michigan; 7 Division of General Medicine, University of Michigan Medical School, Ann Arbor, Michigan; 8 Pathology and Laboratory Medicine Service, Veterans Affairs Ann Arbor Healthcare System, Ann Arbor, Michigan; 9 Department of Pathology, University of Michigan Medical School, Ann Arbor, Michigan; 10 Pulmonary and Critical Care Medicine Section, Medicine Service, Veterans Affairs Ann Arbor Healthcare System, Ann Arbor, Michigan; 11 Division of Pulmonary and Critical Care Medicine, University of Michigan Medical School, Ann Arbor, Michigan; 12 Office of the Director, Veterans Affairs Ann Arbor Healthcare System, Ann Arbor, Michigan; 13 Infectious Disease Section, Medicine Service, Veterans Affairs Ann Arbor Healthcare System, Ann Arbor, Michigan; 14 Division of Infectious Disease, University of Michigan Medical School, Ann Arbor, Michigan

## Abstract

**Objective:**

The seroprevalence of severe acute respiratory syndrome-coronavirus-2 (SARS-CoV-2) IgG antibody was evaluated among employees of a Veterans Affairs healthcare system to assess potential risk factors for transmission and infection.

**Methods:**

All employees were invited to participate in a questionnaire and serological survey to detect antibodies to SARS-CoV-2 as part of a facility-wide quality improvement and infection prevention initiative regardless of clinical or nonclinical duties. The initiative was conducted from June 8 to July 8, 2020.

**Results:**

Of the 2,900 employees, 51% participated in the study, revealing a positive SARS-CoV-2 seroprevalence of 4.9% (72 of 1,476; 95% CI, 3.8%–6.1%). There were no statistically significant differences in the presence of antibody based on gender, age, frontline worker status, job title, performance of aerosol-generating procedures, or exposure to known patients with coronavirus infectious disease 2019 (COVID-19) within the hospital. Employees who reported exposure to a known COVID-19 case outside work had a significantly higher seroprevalence at 14.8% (23 of 155) compared to those who did not 3.7% (48 of 1,296; OR, 4.53; 95% CI, 2.67–7.68; *P* < .0001). Notably, 29% of seropositive employees reported no history of symptoms for SARS-CoV-2 infection.

**Conclusions:**

The seroprevalence of SARS-CoV-2 among employees was not significantly different among those who provided direct patient care and those who did not, suggesting that facility-wide infection control measures were effective. Employees who reported direct personal contact with COVID-19–positive persons outside work were more likely to have SARS-CoV-2 antibodies. Employee exposure to SARS-CoV-2 outside work may introduce infection into hospitals.

Severe acute respiratory syndrome-coronavirus-2 (SARS-CoV-2) is a novel coronavirus that emerged in December of 2019 and quickly became a global pandemic. The rapid global spread of SARS-CoV-2 may have been facilitated by unrecognized asymptomatic infection along with high viral load early in the course of infection before people become ill. How the virus is transmitted in the workplace or how that spread might be prevented is not clear; this information is critical to devise optimal infection prevention strategies to protect frontline healthcare workers and patients during the current pandemic and to prevent future outbreaks.

Early in the pandemic in the spring of 2020, the state of Michigan had the third highest incidence of coronavirus infectious disease 2019 (COVID-19) in the United States. Access to COVID-19 testing by reverse transcriptase-polymerase chain reaction (RT-PCR) during this time was limited to symptomatic individuals, so rates of infection in asymptomatic or minimally symptomatic employees have been difficult to determine. Although healthcare providers (HCP) have had better access to diagnostic testing then the general public, there are still many questions about SARS-CoV-2 infection risk in this population. Recently, serologic assays with high sensitivity and specificity have become available.^1^

Several serologic studies of frontline HCP in the first months of the pandemic demonstrate a wide range of seropositivity. Rates of seropositivity vary greatly across healthcare systems from 36% in New York City,^2^ 44% in London^3^ and 3.8%–17% in China.^4,5^ The reasons for such variable rates of infection in HCP are unclear, but they may reflect the underlying community prevalence as opposed to an increased risk in the hospital. Few studies have looked at HCP across a healthcare system that included staff with both direct patient care and nonclinical functions.

In this study, we invited all paid employees to participate in a serologic survey and questionnaire at the Veterans Affairs Ann Arbor Healthcare System (VAAAHS) from June 8 to July 8, 2020, after the first wave of SARS-CoV-2 infection during March through May 2020. We assessed risk for SARS-CoV-2 seroconversion in VA employees in the healthcare system across multiple exposure settings.

## Materials and methods

### Setting/Population

The VAAAHS is a tertiary-care referral facility within the VA healthcare system that encompasses 5 community-based outpatient clinics. It serves veteran patients in Michigan and Ohio and employs ~2,900 staff.

### Infection control measures

**Fig. 1.**
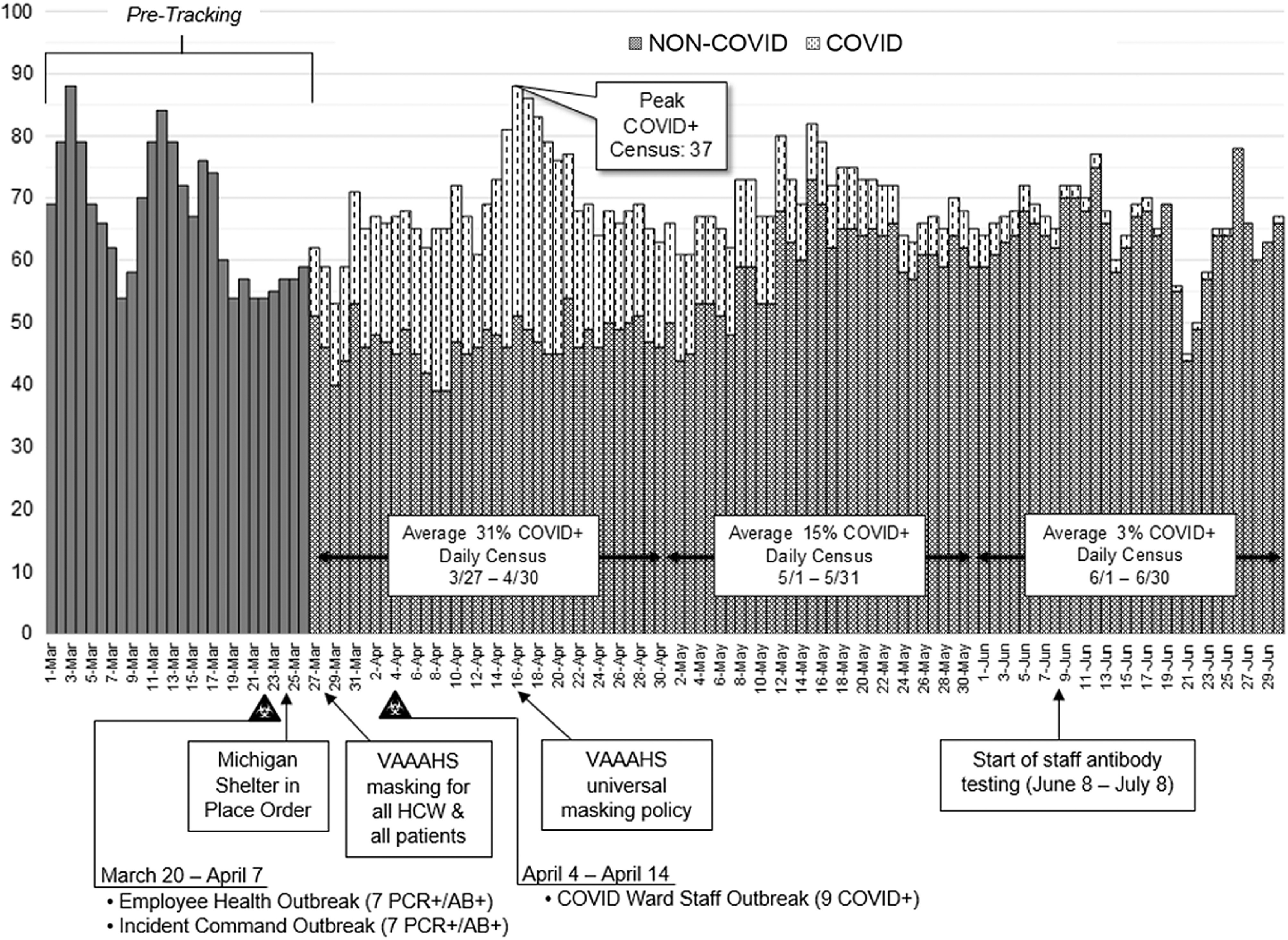
Inpatient daily census and institutional infection control measures for coronavirus infectious disease 2019 (COVID-19). We indicate 3 outbreaks in staff members: 2 identified between March 20 and April 7, 2020, and another between April 4 and April 14, 2020. These known clusters occurred before masking for all persons entering the facility on April 15, 2020.

Multiple infection control measures were instituted, including daily COVID-19 symptom screening upon building entry, exclusion of visitors from the facility, and institution of telework in remote offices or at home (Fig. 1 and Supplemental Table 1 online). All confirmed COVID-19 patients were isolated from non–COVID patients through the creation of 2 intensive care units and on specific inpatient wards, thus maintaining 2 separate zones of care. The COVID-19 wards were converted to negative pressure environments on March 23, 2020, and negative pressure was verified by daily smoke testing. Also on this date, the State of Michigan implemented a stay-at-home order. All personnel who worked on the COVID-19 wards were provided powered air purifying respirators (PAPRs) or N95 respirators along with personal protective equipment (PPE), which consisted of face shields, gowns, and gloves according to the Centers for Disease Control and Prevention (CDC) recommendations in the winter of 2020. Employees who worked on non–COVID-19 wards were required to wear surgical masks while working with patients on March 19, 2020. All other employees providing direct patient care and patients entering the Ann Arbor VA were required to wear a face mask at all times effective March 28, 2020. On April 16, 2020, there was a universal mask requirement for anyone entering the hospital (Fig. 1).

### Serology survey

All VAAAHS employees, exclusive of trainees and volunteers, were eligible for SARS-CoV-2 antibody testing from June 8, 2020, to July 8, 2020. Employees were sent a memo from the medical center director by e-mail about the initiative and how to participate. A second e-mail was sent by a web-based secure and Health Information Portability and Accountability Act (HIPAA)–compliant link (Leaf Software Solutions, Carmel, Indiana), which contained additional information about the study. An opt-in agreement to participate and a link to a brief questionnaire appeared once the employee was enrolled. Employees could choose not to answer the survey or any question. Survey questions focused on the time starting March 1, 2020, 2 weeks prior to the first known COVID-19 case in the health system.

### Questionnaire

Age, gender identity, and county of residence were asked to evaluate for the effects of these variables on antibody response (Table 1). Questions in this survey addressed exposure risk to COVID-19 patients (Table 2). Specifically, the survey addressed the following: whether the employee was involved in direct patient care, specific wards they worked on, and whether they cared for known COVID-19–positive patients. We asked about the presence of COVID-19 symptoms, whether the employee was tested by RT-PCR, and if so, for the result of COVID-19 testing. Participants were also asked whether they had been exposed to known COVID-19 cases outside the workplace. An exposure was defined as close contact (within 2 m or 6 feet) with an individual with confirmed COVID-19 for >15 minutes with the example being exposed to a family member at home who has had a positive COVID-19 nasal swab.

**Table 1.** Demographic Characteristics and Seroprevalence of 1476 Employees Tested for IgG Antibody

Variable	Total, No. (%)	Seropositive, No. (%)	Odds Ratio	95% CI	*P* Value
**Gender Identity**					
Female	944 (64.0)	47 (5.0)	1 [Ref.]	…	…
Male	489 (33.1)	23 (4.7)	0.94	0.57–1.57	.82
No response	43 (2.9)	2 (4.7)	…	…	…
**Age group, y**					
18–30	111 (7.5)	8 (7.2)	1 [Ref.]	…	…
31–40	372 (25.2)	11 (3.0)	2.55	1.00–6.50	.05
41–50	392 (26.6)	17 (4.3)	1.71	0.72–4.08	.22
51–60	406 (27.5)	25 (6.2)	1.18	0.52–2.70	.69
>60	149 (10.1)	8 (5.4)	1.37	0.50–3.77	.54
No response	46 (3.1)	3 (6.5)	…	…	…
**County of residence (only listed counties for >100 participants)**					
Washtenaw (MI)	480 (32.5)	22 (4.6)	1 [Ref.]	…	…
Wayne (MI)	308 (20.9)	21 (6.8)	1.52	0.82–2.82	.18
Livingston (MI)	113 (7.7)	7 (6.2)	1.38	0.57–3.30	.48
Other	415 (28.1)		…	…	…
No response	160 (10.8)		…	…	…

Note. CI, confidence interval; MI, Michigan.

**Table 2.** Occupational and Community Exposure Characteristics and Seroprevalence of SARS-CoV-2 in Veterans Affairs Medical Center Employees

Variable	Total, No. (%)	Seropositive, No. (%)	Odds Ratio	95% CI	*P* Value
**Job description**					
Advanced care practitioner	214 (14.5)	8 (3.7)	1 [Ref]	…	…
Administrative	357 (24.2)	15 (4.2)	1.13	0.47–2.71	.79
Clinical support (direct patient care)	311 (21.1)	10 (3.2)	0.86	0.33–2.20	.75
Clinical support (no direct patient)	83 (5.6)	7 (8.4)	2.37	0.83–6.76	.11
Nursing	412 (27.9)	29 (7.0)	1.95	0.88–4.34	.10
No response	99 (6.7)	3 (3.0)	…	…	…
**Provision direct patient care**					
No	628 (42.6)	32 (5.1)	1 [Ref]	…	…
Yes	814 (55.2)	39 (4.8)	0.94	0.58–1.51	.79
No response	34 (2.3)	1 (2.9)	…	…	…
**Work location**					
No patient care	683 (46.3)	33 (4.8)	1 [Ref]	…	…
COVID ward (includes ED)	388 (26.3)	22 (5.7)	1.18	0.68–2.06	.55
Patient care; non-COVID Ward	243 (16.5)	8 (3.30)	0.67	0.31–1.47	.32
Did not answer	162 (11.0)	9 (5.6)	…	…	…
**Entry COVID-19 patient room**					
No	1067 (74.0)	49 (4.6)	1 [Ref]	…	…
Yes	375 (26.0)	22 (5.9)	1.29	0.77–2.17	.33
No response	34 (2.3)	1 (2.9)	…	…	…
**Aerosol-generating procedure**					
No	1291 (87.5)	66 (5.1)	1 [Ref]	…	…
Yes	155 (10.5)	5 (3.2)	0.62	0.25–1.56	.30
No response	30 (2.0)	1 (3.3)	…	…	…
**Direct exposure to a known COVID-19 case outside work**					
No	1296 (87.8)	48 (3.7)	1 [Ref]	…	…
Yes	155 (10.5)	23 (14.8)	4.53	2.67–7.68	<.0001
No response	25 (1.7)	1 (4.0)	…	…	…

Note. SARS-CoV-2, severe acute respiratory syndrome coronavirus-2; CI, confidence interval; ED, emergency department; COVID-19, coronavirus infectious disease 2019; Ref, reference category.

### Antibody testing

Serum IgG to the nucleoprotein of SARS-CoV-2 was measured using a Federal Food and Drug Administration (FDA) emergency-use–authorized chemiluminescent microparticle immunoassay performed on an automated high throughput chemistry immunoanalyzer (Architect i2000SR, Abbott Laboratories, Abbott Park, IL). Results are reported in a relative light units (RLU) index; a value ≥1.4 RLU is considered a positive antibody response. Though not a direct titer, higher index values highly correlate to neutralization titers.^6^ The sensitivity of this assay is reported to be 100% with a specificity of 99% at >14 days after symptom onset in those infected with SARS-CoV-2.^1^ At 5% prevalence, the positive predictive value is 93.4% and the negative predictive value is 100%.

### RT-PCR testing

Some employees were tested for SARS-CoV-2 infection by RT-PCR during an outbreak investigation. Nasopharyngeal swabs were collected for RT-PCR SARS-CoV-2 testing on employees suspected of having active infection and placed in universal transport media. RT-PCR was performed using the Cepheid GeneXpert platform (Sunnyvale, CA) which amplifies the SARS-CoV-2 N and E genes. Additionally, some employees had testing performed at other facilities and reported results to the employee health department.

### Human subjects

Our study protocol was reviewed by the VAAAHS Institutional Review Board. The study was deemed exempt from approval, and informed consent was waived based on the use of existing HIPPA-deidentified data.

### Data analysis

COVID-19 seropositivity and its associations with categorical variables (eg, age range, gender identity, exposure to COVID-19 patients, job description, hospital ward, and county of residence) were assessed by χ^2^ analysis. Univariate and multiple logistic regression were used to evaluate for factors significantly associated with antibody to SARS-CoV-2. Odds ratios and corresponding 95% confidence intervals were estimated. Statistical significance was determined at *P* < .05. All statistical analyses were performed using Statistical Analysis Software (SAS Institute, Cary, NC).

## Results

Of the ~2,900 eligible employees, 51% participated in the study and 72 of 1,476 (4.9%) were SARS-CoV-2 seropositive (95% CI, 3.8%–6.1%). Furthermore, 21 participants did not answer any survey questions (1.4%). Most respondents were women (64%), and 43 (2.9%) did not answer this question. There were no differences in seroprevalence based on gender or age range (Table 1).

The rate of COVID-19 infection in southeastern Michigan during the first half of 2020 varied based on county of residence (Supplementary Table 2 online). Wayne County had the highest infection rate based on RT-PCR in the state in comparison with Washtenaw County, the county where the VAAAHS is located. Therefore, we examined whether county of residence affected rates of seropositivity. In our study, the seroprevalence was similar between healthcare workers from both counties; Washtenaw County residents showed a seroprevalence of 4.6% and Wayne County showed a seroprevalence of 6.8% (OR, 1.52; 95% CI, 0.82–2.82); this information is similar to published cumulative prevalences for Washtenaw County (3.2%) and Wayne County (5.3%) as of July 8, 2020 (Table 1 and Supplementary Table 2 online).

The seroprevalence did not significantly differ among groups with direct patient care versus those without, or among those working on COVID-19 wards versus non-COVID wards (Table 2). In our study, 814 (55.2%) reported direct patient contact, 628 (42.6%) had no patient contact, and 34 (2.3%) did not respond.

Of the participants in our study, 412 (27.9%) were nursing staff (registered nurses, licensed practical nurses, and clinical nurse specialists and nursing aides); 214 (14.5%) were advanced-care practitioners (physicians, dentists, nurse practitioners, physician assistants, and nurse anesthetists); 357 (24.2%) were administrative or clerical staff; 311 (21.1%) were clinical support staff with patient contact; 83 (5.6%) were clinical operations support services with no patient contact; and 99 (6.7%) did not answer the job question (Table 2).

We evaluated whether job title was associated with higher risk of seroconversion and found no significant difference. The performance of aerosol-generating procedures also did not confer any significant effect on seropositivity (Table 2). There was a statistically significant effect on SARS-CoV-2 seropositivity for those who reported an exposure outside the healthcare system. The seroprevalence with reported exposure outside the healthcare system was 14.8% (23 of 155) compared to only 3.7% (48 of 1,296) without reported outside exposure (OR, 4.53; 95% CI, 2.67–7.68; *P* < .0001). When this analysis was adjusted for working on a COVID-19 ward, outside exposure remained a significant risk of seroconversion.

**Fig. 2.**
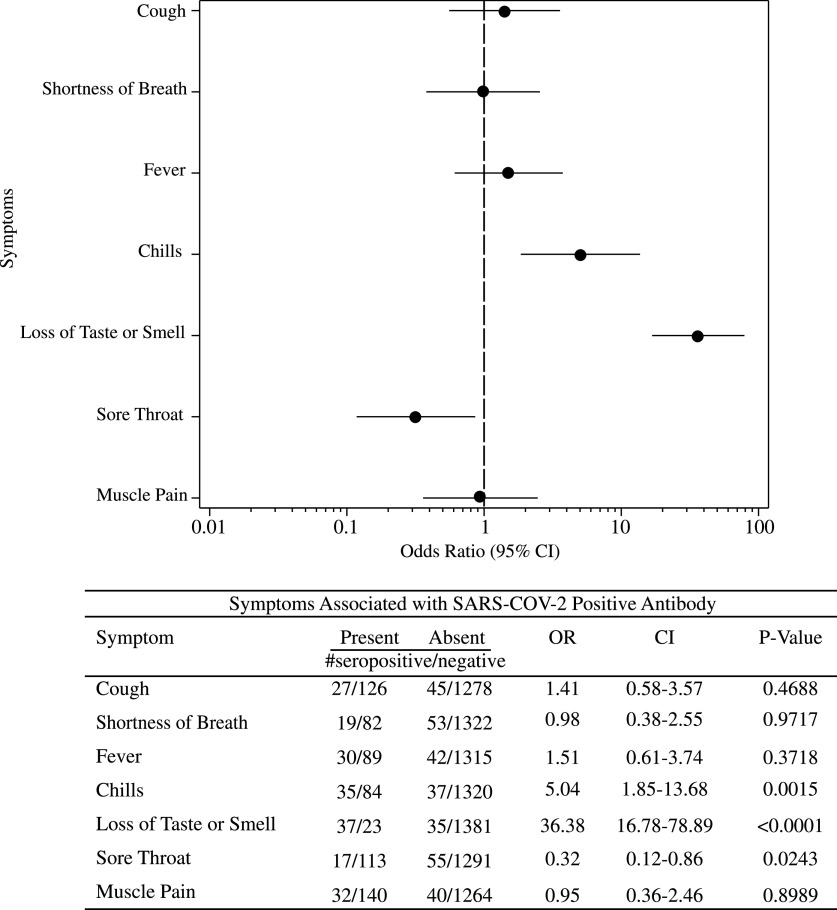
Forest plot showing results of multiple variable logistic regression model of symptoms associated with severe acute respiratory syndrome coronavirus-2 (SARS-CoV-2) seropositivity. For presence and absence of symptoms, we indicate the number of seropositive employees and the number of seronegative employees for each symptom category.

Of 70 participants, 20 (29%) reported that they had no symptoms but were seropositive (2 employees did not answer this question). As expected, employees with symptoms had a higher seroprevalence than those who did not: 50 of 278 (18%) and 20 of 1,170 (1.7%), respectively (OR, 12.61; 95% CI, 7.37–21.59; *P* < .0001). The loss of sense of taste and/or smell had the highest association with seroconversion (OR, 36.38; 95% CI, 16.78–78.89; *P* < .0001) (Fig. 2). Cough, fever, muscle pain, and shortness of breath were not significantly associated with seroconversion. Sore throat was negatively associated with seroconversion (OR, 0.32; 95% CI, 0.12–0.86; *P* = .024).

As of June 8, 2020, 45 employees and 93 patients had tested positive for COVID-19 by RT-PCR at our healthcare system. Of these 45 employees, 5 (11%) were positive before March 30, the date of required masking for all direct patient care personnel and patients, and 33 of 45 (73%) were infected within 14 days after the requirement for universal masking for all persons entering VAAAHS (Fig. 1).

Of the 35 employees who were known to be RT-PCR positive who participated in the serosurvey, 31 (88.6%) also had IgG antibody. Although the cutoff for positivity for this antibody assay is >1.4 RLU, the values for 2 of the 4 RT-PCR positive participants with negative antibody results were >1.15 RLU. The median RLU for all seronegative employees in the survey was 0.03 RLU, suggesting that at least 2 of the 4 infected employees were likely antibody positive and had a waning immune response. Of 195 participants who had a negative COVID-19 nasal swab for symptoms or contact tracing, 9 (4.6%) were positive for SARS-CoV-2 antibodies, and 4 reported symptoms.

## Discussion

In this study, we evaluated the seroprevalence of SARS-CoV-2 antibodies in a VA healthcare system consisting of both frontline healthcare workers as well as clinical support staff and administrators to assess risk of seroconversion. We found a seroprevalence of 4.9% for employees in our healthcare system. Interestingly, there was no difference in the seroprevalence between frontline workers who had exposure to patients during the pandemic, those who worked on a COVID-19 ward or those who performed aerosol generating procedures compared to those who did not have any of these exposures. Our data support the notion that facility-wide infection control measures were effective in preventing infection among healthcare workers. This finding is further supported by the presence of significantly higher rates of SARS-CoV-2 antibody seroconversion among those who reported exposures outside the healthcare system. We suspect that many of these infections occurred in the household. Our data are consistent with a recent study from Belgium showing that the risk of seroconversion is higher outside than within the healthcare system where strict infection control measures are in place.^7^ A recent large study in Boston showed that institution of infection control measures was temporally associated with a decrease in the rate of seroconversion.^8^

We expected that healthcare workers, particularly nursing staff, who had greater potential exposure to SARS-CoV-2 would have a higher risk of seroconversion. Nursing staff often enter COVID-19 patient rooms numerous times per shift to deliver care, whereas advanced practice providers do not. In our sample, nurses had the highest seroprevalence of 7.0%, but this was not significantly different from individuals in other job descriptions. This finding was similar to that in a recent study of healthcare workers in Houston, Texas.^9^ We suspect that the reason for the trend toward greater rates of seroconversion in nursing staff is not related to exposure to patients but exposure in other areas of the hospital to infected coworkers. Contact tracing of clusters of infection within ward personnel revealed that several of the infected nurses shared a break area, eating meals in close contact without masking. We also had 2 additional outbreaks, one in hospital administrative staff working on the same floor and another in the employee health department. All of these outbreaks occurred before the implementation of universal masking in the facility (Fig. 1 and Supplementary Table 1 online). These small outbreaks support the idea that infections occur in groups where masks are not worn.

Our overall rate of seroconversion was similar to a study in a tertiary-care center in Belgium that demonstrated a 5%–6% seropositivity rate but was significantly lower than that found in other studies of tertiary-care centers. Frontline healthcare workers at an academic hospital in New York City had a seroprevalence of 36% (102 of 285).^2^ In London, the rate of seropositivity in frontline healthcare workers was 44% (87 of 200 workers).^3^ The reason for such large variation in seroprevalence across healthcare systems is unclear but could reflect lack of available PPE early on in the pandemic or the level of seropositivity in the community in which healthcare workers live. For instance, one study of cumulative incidence of SARS-CoV-2 in New York City through March 29, 2020, showed an incidence of 23%.^10^ No PPE shortages occurred at our facility. Washtenaw and Wayne counties, where our VA facility is located and where the largest proportion of study participants reside, reported positive testing rates of 3.27%–5.34% as of July 8, 2020, based on RT-PCR testing. These rates are relatively low compared to other areas such as New York City.

At our healthcare system, we instituted infection control measures that exceeded the early 2020 CDC recommendations by providing N-95 respirators or PAPRs to all workers providing direct care on COVID-19 wards. These measures may provide better protection from SARS-CoV-2 infection than surgical masks, but direct comparisons with surgical masks needs to be conducted.

We found that symptoms predicted seroconversion; however, we also found that 29% of seropositive employees were asymptomatic, confirming that many people do not have symptoms when infected with SARS-CoV-2. As previously reported, the loss of sense of taste or smell was a strong predictor of infection (Fig. 2). Chills were also associated with seroconversion, but interestingly, fever, cough, muscle pain, and shortness of breath were not. Oddly, sore throat was a negative predictor of SARS-CoV-2 seroconversion. These results may reflect the wide variability in symptoms experienced during COVID-19 infection and sampling.

The strengths of our study included a large and wide representation of employees with variable exposure to SARS-CoV-2 at their job and a good survey response rate, and we were able to identify potential exposures outside the workplace as well. Our facility was also located in a state with a relatively high incidence rate at the time of our study. We found a seroprevalence of 4.9% among employees in our healthcare system. There has been concern regarding the performance characteristics of some SARS-CoV-2 antibody tests. With a reported positive predictive value of 93.4% for Abbott SARS-COV-1 IgG testing when prevalence is 5%, there may have been ~4–5 false positives in our study. We know of at least 2 participants that were likely false negatives for antibody testing with positive PCR but antibodies levels just below the positive threshold. We do not believe these few false positives or negatives are numerous enough to change the conclusions of our study given that they are likely evenly distributed across our sample. It is possible that some of the nonsignificant or significant variables that we assessed could be due to insufficient power to detect differences due to low prevalence, underreporting, or due to the voluntary nature of the survey, selection, or recall bias.

The commitment to protecting employees from SARS-CoV-2 infection in the workplace continues. Healthcare workers may have better access to testing and personal protective measures than people who work in other settings. Importantly, our study suggests that infection rates can be minimized in work settings if proper infection control measures are instituted including social distancing, isolating COVID-19 patients, and masking. We have also highlighted the importance of screening employees for direct personal exposure to known SARS-CoV-2 infected persons outside the workplace.

## Supplementary material

For supplementary material accompanying this paper visit http://dx.doi.org/10.1017/ice.2020.1220. click here to view supplementary material
