# Supplementary material for: Seroprevalence of severe acute respiratory syndrome coronavirus-2 (SARS-CoV-2) infection among Veterans Affairs healthcare system employees suggests higher risk of infection when exposed to SARS-CoV-2 outside the work environment
Source: Infect Control Hosp Epidemiol. 2020 Sep 23:1–7. doi: 10.1017/ice.2020.1220 (PMC7578654; doi:10.1017/ice.2020.1220)
Supplement: Supplementary file 1 [file S0899823X20012209sup001.docx]

**Online Supplementary Material (Table 1):**

Timeline of Veterans Affairs Ann Arbor Healthcare System Infection Control Measures:

March – April 2020.

| **Date** | **Interventions Implemented** | **Employee Outbreak Comments** |
| --- | --- | --- |
| 3-11-2020 | Patient symptom telephone screening and on entry  Symptomatic patients masked  No visitors or volunteers allowed in facility |  |
| 3-16-2020 | Incident Command Center established  Pause all non-essential clinical services  Telework initiated |  |
| 3-17-2020 | Staff symptom screening on entry |  |
| 3-19-2020 | Masking direct patient care personnel  COVID/PUI – N95/PAPR  Non-COVID – surgical masks  Droplet precautions if respiratory symptoms |  |
| 3-20-2020  to  4-7/2020 | Incident Command Outbreak  Employee Health Outbreak | RT-PCR (+) = 5  Antibody only (+) = 2*  Antibody (negative ) = 24  RT-PCR (+) = 3  Antibody only (+) = 4*  Antibody (negative) = 8 |
| 3-23-2020 | COVID ICUs (2) and (3) wards established – all negative pressure  Non-COVID ICU (1) and (3) wards – not negative pressure |  |
| 3-24-2020 | State of Michigan Order to Shelter in Place |  |
| 3-28-2020 | Masking- all direct patient care staff and patients |  |
| 4-4-2020  To  4-14-2020 | COVID-19 Ward Outbreak | RT-PCR (+) = 9 nurses**  RT-PCR (-) = 56*** |
| 4-16-2020 | Universal masking for all employees, patients, and for essential visitors |  |

COVID-19= coronavirus infectious disease 2019

PUI = persons under investigation

ICU = intensive care unit

PAPR = powered air purifying respirators

RT-PCR = reverse transcriptase polymerase

* diagnosed retrospectively, met exposure criteria in space and time

** shared breakroom and shifts

***nurses, nursing assistants, hospitalists, clerks, housekeepers

**Online Supplemental Materials: Table 2.**

Veterans Affairs Ann Arbor Healthcare System Employee State and County of Residence:

Number of Cases and Cases per 100,000 population from 3/1/2020 to 7/8/2020

| State/County of Residence | State/County Total Cases N (%) | State/County Cases per 100,000 | Tests positive (%) | Employee  Residence N (%) |
| --- | --- | --- | --- | --- |
|  |  |  |  |  |
| **MICHIGAN** | **71,201** | **712.9*** | **3.53** |  |
| Wayne | 11,434 (16.1) | 653.6 | 5.34 | 308 (20.9) |
| Oakland | 9,795 (13.8) | 778.7 | 4.15 | 92 (6.2) |
| Macomb | 7,786 (10.9) | 890.9 | 5.85 | 9 (0.6) |
| Kent | 5,477 (7.7) | 833.7 | 4.49 | 3 (0.2) |
| Genesee | 2,383 (3.3) | 587.2 | 2.03 | 25 (1.7) |
| Washtenaw | 1,719 (2.4) | 467.6 | 3.27 | 480 (32.5) |
| Saginaw | 1,400 (2.0) | 734.8 | 5.12 | 5 (3.4) |
| Ottawa | 1,261 (1.8) | 432.1 | 3.55 | 1 (.06) |
| Ingham | 1,183 (1.7) | 404.6 | 3.73 | 7 (0.5) |
| Jackson | 652 (0.9) | 411.3 | 0.89 | 48 (3.3) |
| Monroe | 589 (0.8) | 391.4 | 1.71 | 66 (4.4) |
| Livingston | 514 (0.7) | 267.7 | 0.00 | 112 (7.6) |
| Lenawee | 253 (0.4) | 256.9 | 3.62 | 29 (2.0) |
|  |  |  |  |  |
| Not done |  |  |  | 11 |
| Calhoun  Eaton  Hillsdale  Shiawassee | 548  296  196  264 |  |  | 2  2  3  4 |
|  |  |  |  |  |
| **OHIO** | **60,181** | **514.8*** | **6.9** |  |
| Lucas | 2836 (4.7) | 662.1 | Not available | 100 (6.8) |
| Wood | 452 (0.8) | 345.5 | Not available | 20 (1.4) |
|  |  |  |  |  |
| Not done |  |  |  | 1 |
| Henry | 39 |  |  | 1 |
|  |  |  |  |  |
| **No Response** |  |  |  | 160 (10.8) |
|  |  |  |  |  |
| **All Employees** |  |  |  | 1476 (100) |

https://www.michigan.gov (accessed 7/31/2020)

<https://coronavirus.ohio.gov/wps/portal/gov/covid-19/dashboards/overview> (accessed 8/1/2020)

https://usafactsstatic.blob.core.windows.net/public/data/covid-19/covid_county_population_usafacts.csv (accessed 7/31/2020)

* Calculated using 2019 State populations for Michigan (9.987 million) and Ohio (11.69 million)
